# High frequency of the expanded *C9ORF72* hexanucleotide repeat in familial and sporadic Greek ALS patients

**DOI:** 10.1016/j.neurobiolaging.2012.02.021

**Published:** 2012-08

**Authors:** Kin Y. Mok, Georgios Koutsis, Lucia V. Schottlaender, James Polke, Marios Panas, Henry Houlden

**Affiliations:** a Department of Molecular Neuroscience, UCL Institute of Neurology,London, UK; b Neurogenetics Unit, Department of Neurology, University of Athens Medical School, Eginitio Hospital, Athens, Greece; c MRC Centre for Neuromuscular Diseases, UCL Institute of Neurology and The National Hospital for Neurology and Neurosurgery,London, UK

**Keywords:** ALS, C9ORF72, Expansion, Hexanucleotide, Greek population

## Abstract

An intronic expansion of a hexanucleotide GGGGCC repeat in the *C9ORF72* gene has recently been shown to be an important cause of amyotrophic lateral sclerosis (ALS) and frontotemporal dementia (FTD) in familial and sporadic cases. The frequency has only been defined in a small number of populations where the highest sporadic rate was identified in Finland (21.1%) and the lowest in mainland Italy (4.1%). We examined the *C9ORF72* expansion in a series of 146 Greek ALS cases, 10.95% (*n* = 16) of cases carried the pathological expansion defined as greater than 30 repeats. In the 10 familial ALS probands, 50% (*n* = 5) of them carried a pathologically large expansion. In the remaining 136 sporadic ALS cases, 11 were carriers (8.2%). None of the 228 Greek controls carried an expanded repeat. The phenotype of our cases was spinal (13/16) or bulbar (3/16) ALS, the familial cases were all spinal ALS and none of our cases had behavioral frontotemporal dementia. Expansions in the *C9ORF72* gene therefore represent a common cause of ALS in Greece and this test will be diagnostically very important to implement in the Greek population. The frequency is higher than other populations with the exception of Finland and this may be due to Greece being a relatively isolated population.

## 1 Introduction

Amyotrophic lateral sclerosis (ALS) is a progressive neurodegenerative disorder characterized by degeneration of the brain and spinal cord leading to weakness, wasting, bulbar problems, and usually death within 3 years (Clarke et al., 2009). Originally noted in an essay in 1830 by Charles Bell the disorder has been described throughout the world with an ALS incidence of between 1.7 and 8.2 people per 100,000 in populations in Europe and the United States, the lowest incidence being in Italy (Turin) (Leone et al., 1983) and highest in Finland (Beghi et al., 2011; Cronin et al., 2007; Logroscino et al., 2010; Murros and Fogelholm, 1983). Approximately 10% of ALS cases are familial, and genetic defects have been identified in approximately a third of families (Rohrer and Warren, 2011; Valdmanis and Rouleau, 2008; Vance et al., 2006) and a small percentage of sporadic cases, in an ever-increasing number of genes that include: superoxide dismutase 1 (*SOD1*) (Rosen et al., 1993), transactivation response (TAR) DNA binding protein (*TARDBP*) (Sreedharan et al., 2008), fused in sarcoma (*FUS*) (Kwiatkowski et al., 2009), valosin-containing protein (*VCP*) (Johnson et al., 2010), factor-induced gene 4 (*FIG4*) (Chow et al., 2009), angiogenin (*ANG*) (Greenway et al., 2006), ubiquillin *2* (*UBQLN2*) (Deng et al., 2011), and optineurin (*OPTN*) (Maruyama et al., 2010).

More than 10 years ago, a locus for autosomal dominant familial ALS and frontotemporal dementia was identified on chromosome 9p21 (Morita et al., 2006). This region was refined to a common 140 kb haplotype containing 3 genes, *MOBKL2B*, *C9orf72*, and *IFNK* that were extensively investigated (Laaksovirta et al., 2010; van Es et al., 2009). Two research groups identified a pathogenic GGGGCC hexanucleotide repeat expansion in intron-1 of the *C9ORF72* gene as an important cause for both familial and sporadic ALS (DeJesus-Hernandez et al., 2011; Renton et al., 2011). They found the expansion (greater than 30 repeats) was present in 46.4% of familial ALS and 21% of sporadic ALS cases from Finland and between 28.5% and 38% familial ALS in series from Italy, Germany, and the United States (Orr, 2011). The expanded repeat was absent in US, Italian, and German controls but present at a rate of 0.4% in Finnish controls. In the UK, pathological expansions accounted for 50% (26/52) of familial and 7% (35/496) of sporadic ALS (Cooper-Knock, 2012). Further analysis of the incidence in sporadic ALS suggests a marked difference across Europe (north to south), the highest prevalence being in Finland (21.1%), then the UK (7%), then Germany (5.2%), Belgium (5%), and the lowest in Italy at 4.1% (Cooper-Knock, 2012; DeJesus-Hernandez et al., 2011; Gijselinck et al., 2012; Renton et al., 2011; Traynor, B.J., personal communication, *C9ORF72* hexanucleotide repeat expansion in sporadic ALS and FTD around the world, 2012). Sardinia is likely to have a higher prevalence, but this is an unusual isolated population which also contains a high frequency of *TARDBP* mutations (Chiò et al., 2011). The overall incidence of ALS may suggest a founder expansion, disseminated by a traveling population such as the Vikings or it could be accounted for by population-specific effects such as isolation. Clinically, the *C9ORF72* expansion causes a frontotemporal dementia (FTD), and an ALS with phenotype is often associated with cognitive problems compared with unexpanded patients. Neuropathologically, abnormal subcellular localization and aggregation of TAR DNA binding protein 43 (TDP-43) is found widely distributed in most patients with ALS and FTD, cortical lesions are TDP-43 positive but cerebellar lesions are negative (Al-Sarraj et al., 2011; Ince et al., 2011; Stewart et al., 2012). These findings further highlight the importance of TDP-43 in FTD and ALS.

There are no further data available on the frequency and phenotype of *C9ORF72* expansions in other European or Mediterranean populations. We therefore examined the frequency, spectrum, and phenotype of patients with an expanded *C9ORF72* hexanucleotide repeat in a well-defined series of ALS cases and controls from Greece.

## 2 Methods

DNA was extracted from 146 ALS cases and 228 controls consecutively collected from the Neurology Department of the University of Athens, Greece. The study was ethically approved with informed consent from patients taking part who were diagnosed according to the El Escorial criteria (Brooks et al., 2000). We screened the *C9ORF72* GGGGCC expansion in the ALS cohort and the controls using the reversed-prime polymerase chain reaction (PCR) protocol as previously reported (Renton et al., 2011). Briefly, 100 ng of genomic DNA was amplified with a final volume of 20 μL containing 10 μL FastStart PCR Master-Mix (Roche, Foster City, CA, USA), 0.18 mM 7-deaza dGTP, 1xQiagenQ solution, 10% dimethyl sulfoxide (DMSO), 0.7 μM reverse primer consisting of 4 GGGGCC repeats and an anchor tail (TACGCATCCCAGTTTGAGACGGGGGCCGGGGCCGGGGCCGGGG), 1.4 μM 6FAM-fluorescent-primer located 280 base pairs 3′ prime to the repeat sequence (AGTCGCTAGAGGCGAAAGC) and 1.4 μM anchor primer corresponding to the anchor tail of the reverse primer (TACGCATCCCAGTTTGAGACG). A touchdown PCR cycling protocol was used where the initial annealing temperature was lowered from 70 °C to 56 °C in 2 °C increments and a 3-minute extension time for each cycle (Paisán-Ruiz et al., 2012). Fragment length analysis was performed on an ABI 3730XL genetic analyzer (Applied Biosystems, Inc., Foster City, CA, USA), analyzed using ABI GeneScan v 3.7 (Applied Biosystems, Inc., Foster City, CA, USA).

## 3 Results

**Fig. 1:**
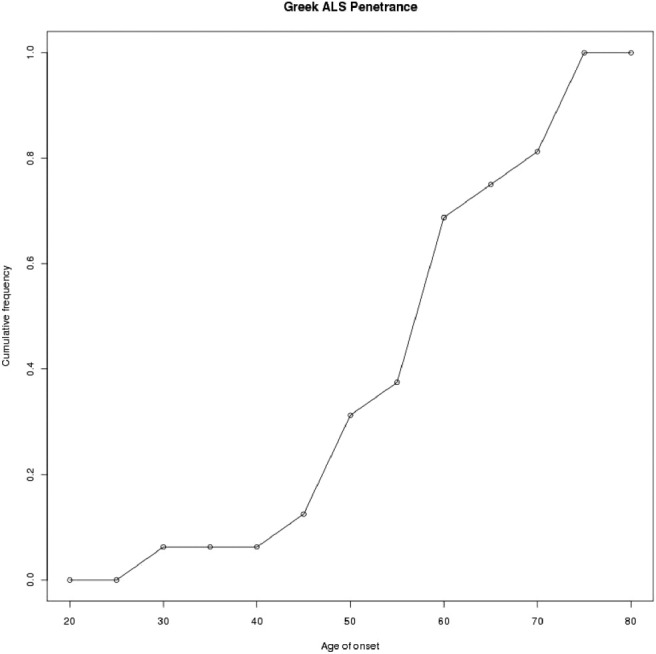
Cumulative frequency of the age of onset in *C9ORF72* expansion carriers.

Overall, 10.95% (*n* = 16) of ALS cases carried the *C9ORF72* pathological expansion (defined as > 30 repeats). In the 146 ALS cases, 10 were familial ALS probands, 50% (*n* = 5) of them carried a pathologically large expansion. In the remaining 136 sporadic ALS, 11 were carriers (8.2%) (Table 1). There was no significant difference in gender, age, and age/site of onset between expansion carriers and noncarriers; there was also no significant difference in the age of onset between patients with and without expansions (Table 1, Fig. 1). No expansions were seen in controls. The cumulative frequency on the age of onset suggested that penetrance is about 50% at 55 years old and almost complete penetrance at 75 (Fig. 1). The phenotype of the familial and sporadic ALS cases was either spinal upper or lower limb onset (13/16) or bulbar (3/16) onset, the age of onset ranged between 25 and 73 years. In familial ALS, there were no bulbar onset cases and a greater preponderance of female cases (Table 1 and Supplementary Table 1).

## 4 Discussion

The identification of *C9ORF72* expansions in ALS and FTD has been an extremely important discovery that will form an essential common diagnostic test in ALS and FTD and also strengthen the link to TDP-43 (Orr, 2011). We report the prevalence of the *C9ORF72* repeat expansion in a cohort of Greek ALS patients revealing a relatively high prevalence of expanded repeats in the Greek sporadic ALS population (8.2%). This is the highest sporadic figure outside of Finland reported to date. These data support the pathological expansion as an important cause of ALS that extends to Mediterranean populations. A further interesting point is that the majority of Greek cases with expansions have a spinal onset, much higher than in the previous reports where the proportion of bulbar onset cases was much higher (DeJesus-Hernandez et al., 2011; Renton et al., 2011).

The 50% prevalence of the expansion within Greek familial ALS is congruent with previous reports. Previously we have reported the likely common founder effect originating from Scandinavia. Haplotypes in the ALS cases from other populations (USA and Italy) also carry part of the Finnish haplotype and it would be extremely interesting to further define the haplotype that harbors the mutation in the Greek population. The high frequency of expansions in the Greek population is not consistent with the reported decreasing frequency of expanded repeats in ALS when traveling south across Europe where frequencies consist of Finland (21.1%), UK (7%), Germany (5.2%), Belgium (5%), and Italy at 4.1% (Cooper-Knock, 2012; DeJesus-Hernandez et al., 2011; Gijselinck et al., 2012; Renton et al., 2011; Traynor, B.J., personal communication, *C9ORF72* hexanucleotide repeat expansion in sporadic ALS and FTD around the world, 2012). These frequencies are similar to the overall distribution of ALS in these countries. It has been suggested the ALS haplotype could be accounted for by the Vikings, but Viking influence was limited in Greece. A more plausible explanation is the relatively isolated nature of the Greek population over recent centuries with low values of past and present racial admixture. This explanation may underlie the high frequency of the ALS haplotype in Finland, which also has a relatively isolated population and a high frequency of ALS and the *C9ORF72* expansion. The high frequency of expansions in apparently sporadic individuals is difficult to explain but is likely due to lack of family history information or previous generations dying early before the expansion caused disease. Another explanation is reduced penetrance of the expansion, and a further question is whether this pathogenic expansion goes through an intermediate expansion/anticipation phase as in other repeat expansion disorders such as Friedreich's ataxia (Orr and Zoghbi, 2007). Although we detected a small number of cases with 20–24 repeats (4/228 = 1.8%) in the control Greek population this frequency is not significantly different when compared with 2 UK control series (unpublished 1/85 = 1.2% and 6/361 = 1.7%).

In summary, we have shown that the pathogenic GGGGCC hexanucleotide repeat expansion in *C9ORF72* is an important cause of ALS in the Mediterranean Greek population in the familial and sporadic forms of disease. The frequency is high, second only to the Scandinavian population and this suggests the repeat prevalence in Greece, and likely in the Scandinavian countries is a consequence of population isolation and little racial admixture. The frequency also highlights the importance of establishing *C9ORF72* as a diagnostic test in familial and sporadic patients.

## Disclosure statement

The authors report no conflicts of interest.

This work was ethically approved, and participating patients provided informed consent.

## Figures and Tables

**Table 1:** Demographic and *C9ORF72* repeat data from the Greek ALS cases

	Total cohort	Familial	Sporadic	*p*	Expanded	Nonexpanded	Expanded (familial)	Expanded (sporadic)
*n* (%)	146	10 (6.8)	136 (93.2)		16 (11.0)	130 (89.0)	5 (50.0)	11 (8.1)
Age as of 01/01/12 (y ± SD)	59.1 ± 13.0	50.8 ± 12.2	59.7 ± 12.9	0.035 a	56.4 ± 12.2	59.5 ± 13.1	57.2 ± 12.6	56.1 ± 12.6
Male	108 (74.0)	4 (40.0)	104 (76.5)	0.020 b	12 (75.0)	96 (73.8)	2 (40.0)	10 (90.9)
Female	38 (26.0)	6 (60.0)	32 (23.5)		4 (25.0)	34 (26.2)	3 (60.0)	1 (9.1)
Age at onset of ALS (y ± SD)	57.3 ± 13.0	49.7 ± 11.1	57.9 ± 12.9	0.055^a^	55.1 ± 12.1	57.6 ± 13.1	55.6 ± 12.0	54.8 ± 12.7
Mode of onset (%)								
Spinal	106 (75.2)	10 (100.0)	96 (73.3)	0.067 b	13 (81.2)	93 (74.4)	5 (100.0)	8 (72.7)
Bulbar	35 (24.8)	0 (0.0)	35 (26.7)		3 (18.8)	32 (25.6)	0	3 (27.3)
*C9ORF72* repeat (%)								
Expanded	16 (11.0)	5 (50.0)	11 (8.1)	0.002 b				
Nonexpanded	130 (89.0)	5 (50.0)	125 (91.9)					

Missing data from 5 sporadic patients. Key: ALS, amyotrophic lateral sclerosis.

a *t* test, familial versus sporadic.

b Fisher exact test, 2-sided *p* value, familial versus sporadic; using a *t* test there was no significant difference seen between expanded versus nonexpanded age at onset.
