# Supplementary material for: High frequency of the expanded C9ORF72 hexanucleotide repeat in familial and sporadic Greek ALS patients
Source: Neurobiol Aging. 2012 Aug;33(8):1851.e1–5. doi: 10.1016/j.neurobiolaging.2012.02.021 (PMC3657168; doi:10.1016/j.neurobiolaging.2012.02.021)
Supplement: Supplementary Table 1 [file mmc1.doc]

**Supplementary table. Demographic and clinical features of *C9ORF72* positive Greek ALS cases**

| **Case number** | **Family history** | **Sex** | **Age (yrs)** | **Age at onset (yrs)** | **Mode of onset** | **EMG findings** | **Clinical features** |
| --- | --- | --- | --- | --- | --- | --- | --- |
| 1 | Sporadic | M | 66 | 65 | Spinal | Denervation on 3 levels | Onset in UL progressing to LL and then bulbar region; signs of UMN and LMN dysfunction in bulbar region, UL and LL |
| 2 | Sporadic | M | 58 | 57 | Spinal | Denervation on 3 levels | Onset in UL progressing to LL and bulbar region; signs of UMN and LMN dysfunction in bulbar region, UL and LL |
| 3 | Sporadic | M | 60 | 58 | Spinal | Denervation on 3 levels | Onset in LL progressing to UL and then bulbar region; signs of UMN and LMN dysfunction in bulbar region, UL and LL |
| 4 | Familial (AD) | F | 75 | 73 | Spinal | Denervation on 3 levels | Onset in LL progressing to UL; signs of UMN and LMN dysfunction in UL and LL |
| 5 | Sporadic | M | 58 | 56 | Spinal | Denervation on 3 levels | Onset in LL progressing to UL; signs of UMN and LMN dysfunction in UL and LL |
| 6 | Familial (AD) | F | 62 | 61 | Spinal | Denervation on 3 levels | Onset in UL progressing to LL and then bulbar region; signs of UMN in bulbar region, UL and LL; signs of LMN dysfunction in UL and LL |
| 7 | Sporadic | M | 27 | 25 | Spinal | Denervation on 3 levels | Onset in UL progressing to LL; signs of UMN and LMN dysfunction in UL and LL |
| 8 | Sporadic | F | 71 | 70 | Spinal | Denervation on 3 levels | Onset in UL progressing to bulbar region and then LL; signs of UMN and LMN dysfunction in bulbar region, UL and LL |
| 9 | Sporadic | M | 46 | 45 | Bulbar | Denervation on 3 levels | Onset in bulbar region progressing to UL and LL; signs of UMN dysfunction in UL and LL; signs of LMN dysfunction in bulbar region, UL and LL |
| 10 | Sporadic | M | 49 | 48 | Spinal | Denervation on 3 levels | Onset in LL progressing to UL and bulbar region; signs of UMN and LMN dysfunction in bulbar region, UL and LL |
| 11 | Sporadic | M | 57 | 55 | Bulbar | Denervation on 3 levels | Onset in bulbar region progressing to UL and LL; signs of UMN and LMN dysfunction in bulbar region, UL and LL |
| 12 | Sporadic | M | 55 | 54 | Spinal | Denervation on 3 levels | Onset in LL and UL progressing to bulbar region; signs of UMN in bulbar region, UL and LL; signs of LMN dysfunction in UL and LL |
| 13 | Sporadic | M | 71 | 70 | Bulbar | Denervation on 3 levels | Onset in bulbar region progressing to UL and LL; signs of UMN and LMN dysfunction in bulbar region, UL and LL |
| 14 | Familial (AD) | F | 47 | 46 | Spinal | Denervation on 3 levels | Onset in LL progressing to UL; signs of UMN and LMN dysfunction in UL and LL |
| 15 | Familial (AD) | M | 56 | 55 | Spinal | Denervation on 3 levels | Onset in LL progressing to UL and then bulbar region; signs of UMN in UL and LL; signs of LMN dysfunction in bulbar region, UL and LL |
| 16 | Familial (AD) | M | 45 | 43 | Spinal | Denervation on 3 levels | Onset in LL progressing to UL and then bulbar region; signs of UMN and LMN dysfunction in bulbar region, UL and LL |

Supplementary table. Legend: AD: autosomal dominant; UL: upper limbs; LL: lower limbs; UMN: upper motor neuron; LMN: lower motor neuron
